# Senior orienteering athletes as a model of healthy aging: a mixed-method approach

**DOI:** 10.1186/s12877-015-0072-6

**Published:** 2015-07-08

**Authors:** Lina Östlund-Lagerström, Karin Blomberg, Samal Algilani, Magnus Schoultz, Annica Kihlgren, Robert J. Brummer, Ida Schoultz

**Affiliations:** Nutrition and Physical Activity Research Centre, Faculty of Health and Medicine, School of Health and Medical Sciences, Örebro University, S-701 82 Örebro, Sweden; Nutrition Gut Brain Interactions Research Centre, Faculty of Health and Medicine, School of Health and Medical Sciences, Örebro University, S-701 82 Örebro, Sweden

**Keywords:** Healthy aging, Successful aging, Orienteering, Mixed-method, Older adults

## Abstract

**Background:**

The proportion of individuals reaching an old age is increasing and will, in the near future consume a majority of health care resources. It is therefore essential to facilitate the maintenance of optimal functionality among older adults. By characterizing older individuals experiencing wellbeing, factors important to promote and maintain health through life can be identified. Orienteering is an endurance-running sport involving cross-country navigation, demanding both cognitive and physical skills of its practitioners. In this study we aim to explore a Swedish population of senior orienteering athletes as a potential model of healthy aging.

**Methods:**

We undertook a mixed-method approach using quantitative (i.e. questionnaires) and qualitative (i.e. focus group discussions) methodologies to explore a population of senior orienteering athletes (*n* = 136, median age = 69 (67–71) years). Quantitative data was collected to evaluate health status, assessing physical activity (Frändin-Grimby activity scale (FGAS)), functional wellbeing (EQ-5D-5 L), gut health (Gastrointestinal symptoms rating scale (GSRS)), anxiety and depression (Hospital Anxiety and Depression scale (HADS)) and overall health (Health index (HI)). The data was further compared to reference values obtained from a free-living Swedish population of older adults. Focus group discussions (FGD) were performed as a complement to the quantitative data to facilitate the individuals’ own views on health and physical activity.

**Results:**

The orienteering athletes enrolled in the study reported a significantly better health compared to the free-living older adults (*p* <0.0015) on all questionnaires except HADS. The high health status displayed in this population was further confirmed by the FGD findings, in which all participants declared their engagement in orienteering as a prerequisite for health.

**Conclusions:**

In conclusion our results show that senior orienteering may represent an ideal model in studies of healthy aging. Furthermore, our results show that even though the senior orienteering athletes are well aware of the long-term benefits of physical activity and have practiced the sport from a young age, they particularly point out that their engagement in orienteering is driven by short-term values such as enjoyment and passion. This may be important to consider when introducing public health interventions among the general older population.

**Electronic supplementary material:**

The online version of this article (doi:10.1186/s12877-015-0072-6) contains supplementary material, which is available to authorized users.

## Background

The proportion of individuals in the western world reaching an old age is still increasing and it is estimated that this segment of the population will consume 75 % of the health care resources by the year 2030 [1]. Research to elucidate the factors that may positively affect healthy aging has become a priority for governments and funding agencies. Identifying factors that are of importance to maintain optimal functionality in old age are essential in order to increase the proportion of independent free-living older adults. Optimal functionality is a multidimensional model conceptualizing factors of importance to promote wellbeing and health from the perspective of the older individual, as explored by Algilani et al. [2].

The research area of healthy aging is commonly addressed by a biomedical approach and has so far been dominated by “disease-directed” thinking [3]. The World Health Organization defines health as a state of complete physical, mental and social wellbeing and not merely the absence of disease or infirmity [4], clearly emphasizing the importance of experiencing both subjective health and wellbeing alongside objective health. Hence, knowledge about objective health based on biomedical science will not be sufficient to meet the social and economic challenges arising as a result of the growing older population. The research community needs to adopt wider approaches and implement bio-psychosocial theories [5–7] in order to reach the desired goals of global health and sustainability accompanied by improved public and social health.

The understanding of what characterizes healthy agers from a bio-psychosocial perspective will complement existing knowledge by adding an important holistic approach. At present, a gold standard of markers and determinants of a healthy aging population does not exist [8]. Centenarians are often studied due to their success in reaching a high age, without considering health status and the many disabilities commonly displayed in this population [9]. It is important to acknowledge that healthy aging it is not solely about reaching a high age, but rather about maintaining optimal functionality [2] and experiencing life-satisfaction and meaningfulness in everyday life [10]. It is essential to identify and characterize cohorts of older individuals experiencing wellbeing and health, at their present age, in order to identify factors important in promoting and maintaining health and sustainability through life.

Orienteering is an endurance-running sport involving navigation in diverse terrain with the help of a map and a magnetic compass, demanding considerable cognitive skills and physical endurance of its practitioners. The sport harbors a substantial proportion of aged athletes and is known for its familiar and social atmosphere [10]. Hence, the orienteering sport holds a unique cohort of aged active individuals. In this study we aim to explore and characterize a Swedish population of senior orienteering athletes as a potential model of healthy aging. In addition, the orienteering athletes’ views on health and motives to stay physically active in old age are thoroughly investigated through focus group discussions.

## Methods

### Mixed-method design

We adopted a mixed-method approach comprising both quantitative (i.e. questionnaires) and qualitative (i.e. focus group discussions (FGD)) data collection methodologies [11], in order to provide complementary knowledge about the topic in question. The qualitative framework was based on a qualitative descriptive design [12] using a content analysis [13, 14].

The study was performed at the multidisciplinary Nutrition and Physical Activity Research Centre (NUPARC) at Örebro University and followed an exploratory sequential design, where qualitative data is used to support the interpretation of the primarily quantitative findings [15]. Quantitative and qualitative data were connected, according to the core of the mixed method approach, in order to thoroughly describe the population of older persons competing in orienteering, as a model of healthy aging.

#### Study outline

**Fig. 1:**
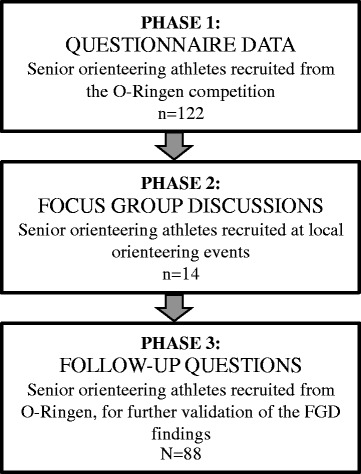
Study overview. Phase 1) An informed consent was sent out to the 200 first competitors over 65 years listed in the O-Ringen registration document. Questionnaires (see Table 1) were sent out to all subjects, prior to O-Ringen. Phase 2) Orienteers attending senior orienteering events in the region of Örebro were asked to file a notice of interest for the study. In total 7 women and 7 men participated in FGDs arranged at Örebro University. Phase 3) Based on the FGD findings a follow-up question was developed and sent out to all participants from phase one, with the intention to validate findings from the FGDs

The study was performed in three phases (see Fig. 1). The first phase of data collection were quantitative and used questionnaires to obtain a cross-sectional evaluation of the health status and wellbeing of a population of orienteers (*n* = 122) participating in the international O-Ringen orienteering event (referred to as OR). The questionnaires, as listed in Table 1, assessed parameters such as gut health and functional health, the level of physical activity and psychological distress. The second phase was a qualitative data collection involving FGDs with female and male orienteers (*n* = 14; 7 women and 7 men) recruited at local orienteering events organized on a regular basis in the region of Örebro (referred to as LO). The FGDs addressed topics about the participants’ perspectives on health and allowed for a deeper understanding of health beliefs and motivation to stay physically active as one becomes older. Based on the findings from the FGDs, a follow-up question was developed (phase 3) in order to quantitatively confirm some of the findings from the obtained qualitative data. The follow-up question was answered by the senior orienteering athletes from phase 1.

**Table 1:** Overview of the included questionnaires

Questionnaire	Variable	Scoring range	
Frändin-Grimby Activity Scale (FGAS) [16]	Measure of habitual physical activity over a whole year.	Estimates physical activity summer and winter using 6 fixed respond alternatives describing different levels of physical activity.	1-6^a^
EuroQol (EQ-5D, EQ-VAS) [18]	Measure of health status.	Five dimensions rated on a 5-point scale using fixed respond alternatives. One question rated on a VAS scale ranging from 0–100.	1-5^a^
			0-100^a^
Health Index (HI) [19]	Measure of general wellbeing, rated on nine questions about energy, temper, fatigue, loneliness, sleep, dizziness, bowel function, pain and mobility.	Nine questions rated on a 4-point scale using fixed respond alternatives.	9-36^a^
Gastrointestinal Symptoms Rating Scale (GSRS) [20]	Measure of gastrointestinal health, constructed by five domains: diarrhea, indigestion, constipation, abdominal pain and reflux.	Fifteen questions rated on a 7-point scale using fixed respond alternatives.	1-7^b^
Hospital Anxiety and Depression Scale (HADS) [21]	Measure of psychological distress divided into two subscales: depression and anxiety.	Fourteen questions (7 questions per subscale) rated on a 4-point scale using fixed respond alternatives.	0-42^b^

^a^High scores are favorable, as this is indicative of health/wellbeing

^b^Low scores are favorable, as this is indicative of health/wellbeing

The study obtained approval from the Uppsala Regional Ethics Review Board (dnr 2012/309). All procedures were carried out in accordance with the Declaration of Helsinki and written informed consent was obtained from all participants.

### Study participants and inclusion criteria

Two populations of senior orienteering athletes were recruited. Participants were included if they were ≥65 years old and actively competing in orienteering. No participants were excluded due to illness or medications, as the aim of the study was to explore and characterize Swedish senior orienteering athletes as a model of healthy aging. Hence, the variation in health and intake of prescribed medication represents an important finding. Sample characteristics are presented in Table 2 and the recruitment process is further described below. The questionnaire data was further compared to reference values from a general older Swedish population (*n* = 238).

**Table 2:** Sample characteristics

	O-Ringen orienteering athletes (OR)	Local orienteering athletes (LO)	Mann Whitney
	(*n* = 122)	(*n* = 14)	*p*-value
Sex, n (%)			
Female	41 (33.6)	7 (50)	
Male	81 (66.4)	7 (50)	
Age, median (IQR)	69 (66–71)	70 (68–72)	0.08
Education^a^, n (%)			
Lower	51 (41.8)	6 (42.9)	>0.9
Higher	71 (58.2)	8 (57.1)	>0.9
Missing	6 (4.8)		
Medicines^b^, n (%)			
BP	23 (18.9)	4 (28.6)	0.48
PPI	8 (6.6)	0 (0)	0.6
Opi	2 (1.6)	0 (0)	>0.9
NSAID	4 (3.3)	0 (0)	>0.9
Yrs of orienteering,			
median (IQR)	36.5 (30–50)	45.5 (31.5-57)	0.12
Smoking, n (%)			
yes	0 (0)	0 (0)	
no	122 (100)	14 (100)	

^a^“Lower education”: NO university studies, “Higher education”: studies at university, “Missing”: non reported

^b^“BP”: Blood pressure medications, “PPI”: Proton pump inhibitors, “Opi”: Opiates, “NSAID”: Non-steroidal anti-inflammatory drugs

### Recruitment of study populations

#### OR (*n* = 122, median age = 69 (66–71) years)

**Fig. 2:**
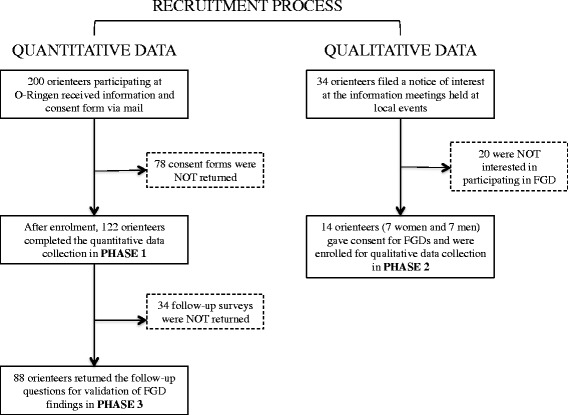
Overview of the recruitment process

Older adults actively practicing and competing in orienteering were recruited based on the start list of the O-Ringen international orienteering event and were invited to complete phase 1 and 3 of the study. See Fig. 2 for an overview of the recruitment process. O-Ringen takes place annually at different locations in Sweden and is the world’s largest orienteering event attracting 15.000–20.000 participants each year. A personal information letter was sent to the first 200 athletes ≥aged 65 years old registered to compete in the 2013 O-Ringen. The letter contained a thorough description of the study as well as a consent form. After signing the consent form the participants were enrolled and given a study code to avoid identification by name throughout the study. The participants were also encouraged to contact the researchers if they had any questions regarding the study.

#### LO (*n* = 14, median age = 70 (68–72) years)

Study participants were recruited from orienteering clubs within the Örebro county for participation in the FGDs (study phase 2). The aim of the FGDs was to qualitatively evaluate the health and beliefs about health among senior orienteering athletes. The small recruitment radius was chosen in order to prevent geographical distance being an obstacle for joining the group discussions. All year, except during the winter season, the orienteering clubs within the Örebro county arrange weekly orienteering competitions solely open for orienteers over the age of 65 years. Informational meetings were arranged in conjunction with some of these orienteering competitions and all orienteers attending were invited to file a notice of interest for participation in this study. Orienteers who notified us for their interest were subsequently contacted via mail with further information concerning the study together with an informed consent.

#### Free-living older adults (*n* = 238, median age = 72.9 (68–75)

Quantitative data was also collected from a general older Swedish population in Örebro county and used as reference values to compare the results obtained from the OR-population.

Free-living older adults were recruited through advertisements placed in local and regional newspaper. Individuals who responded to the advertisement were sent the study information letter by mail and were requested to contact the research team by phone with questions regarding the study before returning the consent form by mail. Each study participant was assigned a contact person (a medical student educated about the study and the parameters used) and subjected to a one-week period of collection of information regarding health status, gastrointestinal discomfort, psychological distress and level of physical activity.

### Data collection

All data was collected from the winter of 2012 until the summer of 2013.

#### Demographic data

The following demographic information was collected in order to better define the study sample: age, sex, education level, marital status, years of orienteering and smoking habits (Table 2).

#### Medications

The use of prescribed medications taken during the 6 months preceding the study start was assessed by self-reporting. Current prescription of medications was documented in the study Case Report Form (CRF). The distribution of prescribed medications is shown in Table 2.

#### Quantitative data–phase 1: OR

The study participants received the questionnaires, as described below, via regular mail and were asked to complete them the week before O-Ringen. Each participant brought the questionnaires to O-Ringen where they were collected by the researchers. A reminder to fill out the questionnaires, together with the time and location for handing them in was sent out by regular mail prior to the event. Background information was collected and reported in the CRF. The researchers checked that the questionnaires had been completed correctly and were also available during the competition to answer questions about the study. The questionnaires used are described below.

##### The Frändin-Grimby Activity Scale (FGAS)

The FGAS was used to evaluate the level of physical activity. The scale states six different scenarios of physical activity and the participants circle the option that best matches their activity level. The scale estimates the activity level over the past year, including a score for both the summer and winter seasons, and has previously been validated among older adults in Sweden [16].

##### EuroQol (EQ-5D-5 L index and EQ-VAS)

EuroQol is a standardized measure of health status and one of the most widely used instruments of its kind worldwide [17]. The tool consists of two parts; part one consists of 5 items (referred to as EQ-5D) related to wellbeing and function: mobility, self-care, usual activities, pain/discomfort and anxiety/depression. Part two consists of a visual analogue scale (referred to as EQ-VAS) recording the respondent’s self-rated health on a vertical scale ranging from 0 to 100 [18].

##### Health Index (HI)

HI measures functional health by asking nine questions related to vigor, temper, fatigue, loneliness, sleep, dizziness, bowel function, pain and mobility. All items are aggregated to a total score ranging from 9 to 36 points. The questionnaire has been developed and validated in Sweden [19].

##### Gastrointestinal Symptoms Rating Scale (GSRS)

Gastrointestinal discomfort was assessed using the GSRS. The reliability and validity of the GSRS are well documented [20]. The scale includes 15 symptoms (e.g. reflux, abdominal pain, indigestion, diarrhea and constipation) scored from 1 to 7 depending on their severity, where score 1 represents “no problems” and score 7 represents “severe problems”. A score of >3 in any of the domains were considered to be moderate gut problems. In this study the total GSRS score was used, as an estimate of overall gastrointestinal discomfort.

##### Hospital Anxiety and Depression Scale (HADS)

HADS was originally developed by Snaith and Zigmond [21] and is a widely used instrument for the evaluation of psychological distress in medical settings. The instrument consists of 14 items, which can either be separated into two subscales measuring anxiety (seven items) and depression (seven items) or can be used as a total score of psychological distress. In the current study the total HADS score was used for the evaluation of psychological distress. The validity and reliability of HADS have been reported in several studies [22].

#### Qualitative data–phase 2: LO

**Fig. 3:**
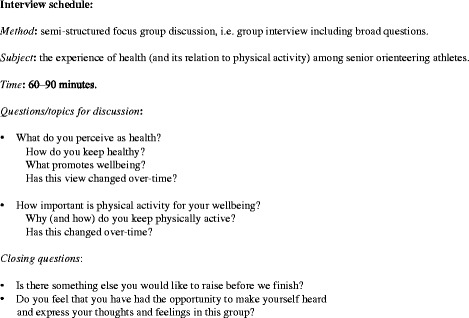
Interview schedule

The method of focus group discussion (FGD) was chosen as it aims to facilitate individuals’ exploration and clarification of their own views through group interaction, in a manner not possible with one-to-one interviews. Each FGD, conducted at Örebro University, Sweden, was led by experienced moderators and followed the guidelines by Barbour & Kitzinger [23]. Thirty-four orienteers expressed their interest in FGD participation and fourteen subjects were able to participate at the assigned date and time. The FGD participants were divided into two focus groups, including 7 female and 7 male orienteers respectively. The recruitment process took place during the fall and winter of 2012/2013. The FGD was semi-structured, including broad questions on the subject of health and physical activity (see interview schedule, Fig. 3). At the beginning of each FGD the moderators emphasized the ethical principal of confidentiality. The role of the moderators was to stimulate the participants to engage in an active discussion and to direct their focus towards the topics of interest. When certain issues were not raised spontaneously, but considered important by the moderators, the moderators initiated discussion on these issues towards the end of the FGD. Each FGD was audio-recorded with the participants’ permission and lasted 60–90 min. All FGDs were transcribed verbatim.

#### Follow-up question–phase 3

A follow-up question was developed and sent to the population of senior orienteers recruited from O-Ringen in order to confirm some of the FGD findings in a larger population. The follow-up survey was constructed as a multiple-choice question asking about the most important reason for being physically active. The alternatives given were 1) “improving my physical health”, 2) “improving my mental health”, 3) “being part of a social context“, and 4) “to perform better at competitions”, where each item was rated on a 4 point scale ranging from “I disagree” to “I fully agree”.

### Data analysis

#### Quantitative data

Median values, presented together with the inter-quartile range (IQR), were generated for all questionnaires and demographic data. Differences in demographic data between OR and LO were analyzed using the Mann–Whitney *U*-test. The same test was also used to compare the orienteering athletes’ data with reference values from a free-living general older population as shown in Table 3. A *p* value of 0.05 was considered to be statistically significant.

**Table 3:** Questionnaire scores from study phase 1, median (IQR)

Questionnaire	OR	Free-living older adults	Mann Whitney
	*n* = 122, age: 69 ± 5 yrs	*n* = 238, age: 72.9 ± 6.5 yrs	*p*-value
FGAS	5 (4–5)	3.5 (3–4)	<0.0001
EQ-5D index	0.86 (0.8-1)	0.86 (0.8-1)	0.0015
EQ-VAS	90 (85–95)	80 (75–90)	<0.0001
HI	31 (29–33)	29 (26–31)	<0.0001
GSRS	1.3 (1.1-1.7)	1.8 (1.3-2.4)	<0.0001
HADS	4 (2–7.3)	4 (3–8)	0.1852
*Depression*	*2 (1–3)*	*2 (1–3)*	
*Anxiety*	*2.5 (1–5)*	*3 (1–5)*	

One questionnaire item (GSRS) had to be imputed due to missing data. The missing value was replaced with the arithmetic mean of the completed items in the questionnaire, in accordance with the instructions for the questionnaire. Eighteen individual questionnaires sheets (FGAS: 9, GSRS: 1, HADS: 5 and HI: 3) had to be excluded from the analysis due to missing values in proportions that did not allow for imputation.

To derive a single EQ-5D index score from the five EuroQol domains, the Danish time-trade-off scoring algorithm was used to weigh each respondent’s profile data [24]. The Danish algorithm was judged as the best available match for our study sample, since no Swedish algorithm has yet been produced.

All statistical analyses were performed using GraphPad Prism version 6 for Mac (GraphPad Software, San Diego California, USA).

#### Qualitative data

The FGDs were transcribed and subsequently analyzed by inductive content analysis using an open coding approach [14]. Two questions were formulated to use as the basis for the analysis: 1) “What is perceived as health and how is health maintained?” and 2) “What contributes to the continuous engagement in physical activity?”. The focus group transcripts were read carefully and notations about meaningful content were made in the margins of the transcript document. Headings were collected from the margins and transferred to a separate coding sheet. “Sub-categories” grouping the headings were freely originated. Subsequently, the generated list of categories was organized under higher order headings, creating so-called “generic categories”. At this step of analysis the data were classified as belonging to a particular group by distinguishing between data that were interpreted as bearing separate meanings. The process of abstraction was concluded with the creation of “Main categories”, i.e. the generic categories were collapsed into broader themes formulating a general description of the content analysis findings. The generic categories were then organized in accordance with the concept of optimal functionality, previously explored and described by our group [2]. The concept is built on three corner-stones 1) self-related factors (e.g. mental wellbeing), 2) body-related factors (e.g. physical wellbeing), and 3) external factors (e.g. demographic and environmental factors). The generic categories were sorted depending on their relation to these three cornerstones, as the present FGD findings were found to correspond well to this theoretical framework.

Three of the authors (K.B., A.K., I.S.) checked and evaluated the resultstables together with the FGD transcripts in order to achieve agreement on the presentation of the qualitative findings and whether these truly reflected the FGD dialogues [14].

## Results

### Background information

Demographic data and information about medications are presented in Table 2. All of the information was collected in the study CRF (*n* = 122), except for the question “years of orienteering” which was collected together with the follow-up question in phase 3. Eighty-eight OR study participants answered the question; result are presented in Table 2, with median and IQR. The populations of senior orienteering athletes (OR and LO) were identified as homogeneous groups according to their similarities in health distribution (Table 2). As outlined in the table none of the orienteering athletes included in the study were smokers and the distribution of medications were found to be similar between the groups. Furthermore, no statistical differences between OR and LO were found on either of the parameters investigated (Table 2). To further support the homogeneity of the study population as well as the representativeness of the FGD sample, all 14 FGD participants reported participation in one or more O-Ringen competitions (median: 18 (12.3–21.3) years).

### Quantitative findings–phase 1:OR

The quantitative findings presented in this section are based on the questionnaire data collected from the OR population in phase 1. All data are compared to reference values obtained from free-living older adults as outlined in Table 3. Out of the 200 invited orienteering athletes 122 completed and returned the consent form, giving a return rate of 61 %.

#### Physical activity level

The median level of physical activity for OR was reported as 5 (4–5) on a 6-graded scale and represents the level of physical activity over the entire year, combining FGAS summer and winter activity scores (Table 3). Six of the 122 OR participants reported a physical activity level of 3, corresponding to a lighter physical activity (e.g. walks, dancing etc.) of 2–4 hours per week. As outlined in Table 3, the senior orienteering athletes reported a significantly higher physical activity level of 5 (4–5), compared to free-living older adults (3.5 (3–4), *p* <0.0001). Furthermore, no difference in activity was observed between the summer and winter season in either of the populations.

#### Health status

**Fig. 4:**
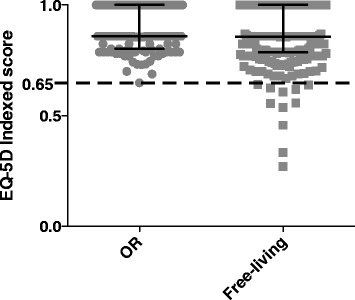
Subjective wellbeing. The Figure shows individually plotted scores for EQ-5D for the two populations in grey together with bars in black indicating median and IQR. The crosshatched line shows an arbitrary cut-off, at 0.65, to help visualize the larger proportion of low scoring subjects among the free-living older adults

The median value of the OR-population for the EQ-5D index was reported at 0.86 (0.80-1), indicating that orienteering athletes experience a high subjective health. The experience of health, as measured by the EQ-5D index, was further found to be significantly higher among senior orienteering athletes compared to free-living older adults (*p* = 0.0015). However, the EQ-5D index median did not differ between the OR population and the free-living older adults. To further show the distribution of the data, the EQ-5D index scores were plotted, as shown in Fig. 4; we identified eleven low scoring subjects among the free-living older adults (4.6 %, 11 out of 238) but none among the OR population.

The experience of a high degree of subjective health among the OR population was further confirmed when the study participants rated their own health on the EQ-VAS (Table 3). The OR participants scored their health significantly higher, (90 (85–95)), than the general population of older adults, (80 (75–90), *p* <0.0001). However, one of the orienteering athletes was found to score low on the EQ-VAS, with a reported score of 50 % of the scale maximum. This was found to correlate with concomitant problems related to pain, dizziness, sleeping, and feelings of fatigue and loneliness on the functional health measure, HI, as well as elevated anxiety levels on the HADS. The distribution of data further showed that 0.82 % (1 of 122) of the orienteering athletes and 13.5 % (32 of 238) of the free-living older adults scored below 70 on the EQ-VAS, further confirming that there are more low scoring subjects among the free-living older adults compared to the senior orienteering athletes.

Moreover, the OR population reported a median score of 31 (29–33) on the HI health measure, indicating a high degree of general wellbeing in this population. This score was found to be significantly higher than the scores reported by the free-living older adults (29 (26–31), *p* <0.0001), suggesting that the free-living population experience more troubles on the domains of energy, temper, fatigue, loneliness, sleep, dizziness, bowel function, pain and mobility.

#### Gut health

The OR population generally reported low scores on the health scale GSRS (1.3 (1.1–1.7)), suggesting an overall good gut function in this population. The frequency of gastrointestinal problems was observed to be significantly lower among the OR population compared to free-living older adults (1.8 (1.3–2.4), *p* <0.0001, Table 3). Moreover, we observed that 42.4 % (*n* = 101) of the free-living older population suffered from moderate gut problems (score >3 on any domain of the GSRS) while only 9.8 % (*n* = 12) of the OR population reported moderate gut problems, suggesting that senior orienteering athletes experience an overall better gut function.

#### Psychological distress

**Fig. 5:**
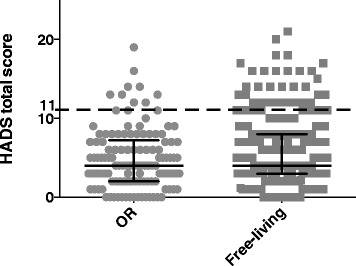
Hospital anxiety and depression scale (HADS). The Figure shows individually plotted scores in grey for the two populations together with bars in black indicating median and IQR, for HADS total scores. The crosshatched line shows a cut-off at eleven, the lowest total score among the OR population. A score ≥8 has been suggested to be indicative of mood disorder

The majority of the OR population displayed low levels of psychological distress, as the median score was 4 (2–7.3) on the physiological wellbeing scale HADS. No significant difference in HADS score was, however, observed between the OR and free-living older adults, indicating that the level of depression and anxiety were equal in the two populations. To further evaluate the distribution of the data, the scores were plotted (Fig. 5); as shown nine senior orienteering athletes reported elevated scores (≥8) on either of the HADS two subscales (7.4 %) compared to free-living older adults, in which 31 participants (13.0 %) reported a high score. A high HADS score was found in the majority of cases to correlate to low scores on the items measuring pain and psychological distress on the EQ-5D index, thus contributing to the low median value. On the HI measure, a high HADS score was found to correspond to a low score on items estimating the study participants’ sleep deprivation, tiredness and pain. Two (1.6 %) of the orienteering athletes identified as scoring high on HADS displayed scores indicative of a probable case of anxiety or depression, as judged by the cut-offs on the two subscales suggested by Snaith [25]. For one of these individuals the high HADS score corresponded to a low score of 50 % of the scale maximum, as previously mentioned.

### Qualitative findings–phase 2: LO

The qualitative findings are presented below in generic categories organized in accordance with the concept of optimal functionality as developed by Algilani et al. [2] (previously described in the qualitative data section). The concept is built on three corner-stones: 1) self-related factors, 2) body-related factors and 3) external factors. Body-related factors refer to health aspects related to the physical function of the body, while self-related factors describe health aspects originating from within one’s self (such as happiness and mental strength) and external factors refer to factors outside of one’s self, for example family and friends. The italic text below corresponds to the generic categories and subcategories identified in relation to each results section and can be found in Tables 4 and 5, as stated in the text.

**Table 4:** Factors contributing to experience and maintenance of health

Main categories	Generic categories	Sub-categories
Body-related factors	Basic hygiene	*Basic hygiene*
	Good gut health	*Getting along with your gut*
		*Good gut health*
	Live the life you desire	*Being able to do the same things as you used to do*
		*Being able to do what ever you want*
		*Being able to go outside*
		*Being able to live the life you used to*
		*Being able to move*
		*Being able to run*
		*Being able to walk*
	Physical activity	*Everyday exercise*
		*Keep Moving*
		*Not being still (not sitting at home)*
		*Physical Activity*
		*To be physically strong*
	Prevention and treating of body-related issues	*Regular health check-ups*
		*To take care of physically related issues*
Self-related factors	Feeling mentally strong	*Feeling mentally stronger than others*
	Having a good time	*Having a good time*
	Keep the brain active	*Keep the brain active*
		*To educate oneself*
	Staying positive to life	*Feeling desire towards moving*
		*Feeling desire towards eating*
		*Keep a positive attitude towards life*
		*Staying positive towards life*
		*Wanting to do things*
External factors	Avoiding un-healthy environments and habits	*Avoiding un-healthy environments (e.g. barrooms) and habits (e.g. drinking, smoking)*
	Being Outdoors	*Being in the forest*
		*Being outdoors*
		*Being outdoors and moving (engaging in physical activity)*
		*Enjoying nature*
		*Enjoying the fresh air*
		*Exercising outdoors*
		*Fresh air and sunshine*
		*Picking berries and mushrooms (in the forest)*
		*Spending a lot of time outdoors*
		*Walking outdoors*
	Cultural engagement	*Experiencing culture*
		*To be a part of the cultural community*
		*To go to the theatre and cinema*
		*To keep up with news*
		*To read books*
		*To read magazines*
		*To travel*
	Healthy diet	*Important to eat well (healthy)*
		*Low fat diet*
		*Making sure that you eat everything that is needed*
		*Not a lot of sugar and cream*
		*Not to be careless with your diet*
		*To think about what you eat*
	Social engagement	*Feelings of kinship*
		*Important to have social exchange*
		*Meeting others*
		*Regular social activities*
		*Social activity without physical activity (going shopping)*
		*Social engagements with the opportunity to exercise*
		*To meet*
		*To socialize*
		*To spend time with friends and family (acquaintances)*
	Social network	*Having a social network*
		*To build a network (of people) for the future*
	Good-tasting food	Good-tasting food

**Table 5:** Factors contributing to continuous engagement in physical activity

Main categories	Generic categories	Sub-categories
Body-related factors	Feeling better than others	*Profit from the physical activity*
		*To feel better than others (physically)*
		*To feel stronger than your peers*
	Good genes	*Good genes (genetic inheritance)*
Self-related factors	Competitiveness	*Being competitive*
		*Being successful*
		*Competing with your self*
		*Orienteering as a sport*
	Interest in sports	*Interest in sports*
	Feeling privileged	*Feeling privileged*
	Feeling talented	*Feeling talented*
	Having fun	*It is like a party*
		*It is fun*
		*To be happy (by physical activity)*
	Passion	*Not a must (driven by will/desire)*
		*Receiving energy (by physical activity)*
	To cope (i.e. to have endurance)	*To keep going (with life)*
		*To keep up a good mood*
External factors	Being outdoors	*Fresh air and sunshine*
		*Near nature and going outdoors*
	Community	*Being part of a community*
	Lifelong sport	*A type of sports that you can be part of for as long as you live*
	Routine	*Getting used to being active*
		*The need to go outdoors for fresh air*
		*The need to move after being still*
	Supportive environment	*Brothers and sisters*
		*Childhood support (family/social)*
		*Childhood sports*
		*Friends*
		*Good PE teachers*
		*Not so many other options (in your youth)*
		*Parents have an important part*
		*Unofficial competitions*
	Social interaction	*Sharing the same interest as your children/partner*
		*Social interaction over generations (lots of different ages)*
		*To socialize*

### Factors contributing to the experience and maintenance of health

This section provides information on the senior orienteering athletes’ experiences of health and how they maintain their health. The different categories and subcategories identified are outlined in Table 4.

#### Body-related factors

The bodily function and the way the individual interprets their own physical fitness directly affected health according to the interviewed senior athletes, and were found to be equally important to both men and women. The senior athletes uniformly stated that being *physically active,* including feeling physically strong and performing outdoor activities, was important for experiencing body related health. The male athletes also expressed that feeling physically strong is advantageous when it comes to interpreting physical health status, for example, one of the athletes disclosed that he did not worry about high cholesterol levels since he knew that he could still perform at a high physical level.

*“I take medications… and apparently I have high levels [of cholesterols] but I’m physically strong. I did two Vasalopp [a 90 km long cross-county skiing race] during the same year, that is Sunday and Sunday [a week between his races], running in orienteering, folk dancing, playing floor hockey and doing core workouts… so I just seem to have high levels [of cholesterols]” (The male focus group)*

The male focus group also discussed the importance of maintaining good *basic hygiene,* such as using antiseptics for cleaning the hands regularly, as well as *preventing and treating body-related issues,* for example by having regular health check-ups at the health-care clinic, as strategies to maintain good physical health. Furthermore, having a well-functioning gut, i.e. a *good gut health,* was described by both the male and female athletes as an important factor to experiencing physical wellbeing. One of the male athletes stated that having a bad gut could largely impact the general experience of physical health and wellbeing.

*“you can see it on anyone that the gut matters, because you don’t have any power and you cant manage anything [if you have a bad gut].” (The male focus group)*

*Living the life you desire* was considered another important aspect in experiencing body-related health and for the senior athletes, this meant being able to move and to exercise, e.g. jogging, skiing, golfing or walking. Exercising outdoors also enabled them to get close to nature, and experience various seasons of the year. Living life as desired also includes being able to continue to live life as usual and engage in physical exercise. The senior athletes further expressed that experiencing bodily health maintained a sense of feeling young.

#### Self-related factors

When discussing self-related health the senior orienteering athletes talked about mental wellbeing, which was referred to as a *feeling of being mentally strong.* The male orienteers remarked that this was a quality that they possessed, particularly in comparison to older people in general. One of the male athletes argued that being physically active also benefit mentally in other aspects of life (i.e. mental aspects).

*“one can endure when others get fed up, ‘What are you whining about?’ It is just pushing forward, and then you just keep on pushing” (The male focus group)*

Among the female orienteers self-related health and mental wellbeing were discussed as being able to *stay positive in life*, meaning maintaining the desire to move and eat, keeping a positive attitude and retaining the will to experience new things. One of the female athletes stated her self-related health as follows:

*“being able to move and walk outside, do whatever you want to do, to wake up and feel ‘oh how nice that it’s a new day’ and you want to do things and you have a positive view of life-that is health to me.” (The female focus group)*

Both the female and male athletes also talked about the importance of *keeping your brain active*, for example by taking university courses or doing crossword puzzles.

#### External factors

The female orienteers stated that eating *good-tasting food* and *experiencing cultural activities* such as going to the theatre, are important for the experience of health*.* Also, *social engagement* was considered vital for experiencing health and wellbeing from an external perspective and the women felt that this actually might be just as important as performing physical exercise. One woman mentioned that she and her friends use a common expression for their way of combining physical activity with social engagement by particularly emphasizing the social aspect.

*“we are calling it ‘social engagement with the opportunity to exercise [referring to their way of performing physical exercise]’”(The female focus group)*

The female orienteers further discussed the importance of *having a social network,* in different areas of life. They all agreed on the importance of expanding one’s social network outside of orienteering in case they are no longer able to continue their active lifestyle.

Furthermore, both the female and male athletes uniformly expressed that being outside in nature was of particular importance for their experience of health and wellbeing.

*“getting out into the forest, that is absolutely necessary. When you get out into that really nice pine forest where it is green on the ground as well and the silence-it is so incredibly fantastic I think, it feels so good”(The female focus group)*

The male orienteers likewise expressed the importance of *social engagement* and *being outdoors* but additionally stated the importance of the environment you spend time in to be health promoting, signifying the importance of avoiding *unhealthy environments and habits* such as visiting smoky bar rooms and drinking alcohol.

### Factors contributing to continuous engagement in physical activity

This section provides information on the senior orienteering athletes’ descriptions of what motives them to stay physically active throughout life. In Table 5 the descriptions of the categories and subcategories are stated in relation to continuous engagement in physical activity.

#### Body-related factors

Body-related factors motivating continuous engagement in physical activity were exclusively discussed by the male athletes, where they, for example, stated *good genes* (i.e. genetic inheritance) as a reason for staying active, meaning that, apart from building a strong body at an early age, staying physically active could also be a consequence of having a genetic predisposition helping one to stay active. They also stated that *feeling better than others* is a motivator to stay active and engage regularly in physical activities. Furthermore, they referred to situations beyond their sport in which they felt they benefitted from being physically active, for example when attending a party.

*“when you are at a party you feel stronger than the others [peers attending the party] because they never move [aren’t physically active], they just sit and fall a sleep.” (The male group)*

#### Self-related factors

The male athletes discussed that performing physical activity needs to be driven by *passion* in order to motivate long-term engagement. The sport you are practicing should bring energy and can’t be “a must”; on the contrary, practicing the sport ought to be driven by will and desire. They also felt that having a general *interest in sports* could be a driver for staying physically active. C*ompetitiveness* was also discussed as a reason for staying engaged in orienteering. They all said that they had a competitive instinct, at least, to some degree. However, solely focusing on competition was not viewed upon as something positive; on the contrary, the fact that you don’t have to win to be appreciated by your fellow sportsmen was thought to be the beauty of orienteering.

*“when you get tired… you can stand still and read the map I think that is the best, as soon as you get tired you can stop and with clean conscious read the map, if you’d run on a court you’d get beheaded or yelled at.” (The male group)*

An additional reason for staying engaged with orienteering was that it gives one the *endurance to cope* with other things in life and improves one’s mood. *Having fun* was also described as an important requisite to staying continuously engaged in your sport; it should make you happy. The women also touched upon the subject of *feeling privileged* to be able to practice their sport, since you can’t take this for granted.

*“you feel privileged to be able to do this [practicing orienteering] since you know how fast life can change” (The female group)*

*Having fun* and *feeling talented* were further described as a self-related motivational factor among the female orienteers.

#### External factors

Both the female and male athletes talked about the significance of growing up in a *supportive environment* (i.e. having a supportive family and friends) as an external factor important in maintaining physical activity over time. Growing up in an environment supporting sports and physical activity provides one with a foundation for staying active throughout life. The supportive elements further discussed by the senior athletes were being surrounded by friends and family that are also engaged in physically activity, having good experiences from the physical education in school, enjoying informal competitions with friends, and the fact that alternative entertainment options were scarce in their youth. One of the female athletes talked about her father being a driving force for her and her cousins and friends when it came to physical activity during school holidays.

*“my father took time off from work when we had the February holiday and then you had to be outside skiing and you had to bring cousins and such, whose parents weren’t as interested, because it was important first and foremost to be outside skiing and moving” (The female group)*

The senior athletes also expressed the importance of *being outdoors,* enjoying nature and sunshine, as a motivating factor for continuing their engagement in orienteering. Also, when the physical activity becomes *a* r*outine,* it becomes an essential part of your life. They all felt the need to be active in order to be satisfied. Two of the female orienteers expressed themselves as follows:

*“especially if you have been sitting in the car for a while, you almost have to go out and move-(perform exercise), it becomes a need” [another woman continues] “I believe you’ve gotten used to it, you get abstinence problems if you can’t move [exercise]” (The female group)*

Both the female and male groups also viewed the opportunity for *social interactions* as a very important motivating factor for continuous engagement in the sport. Orienteering provides plenty of opportunities for socializing, as one often shares the interest in the sport with family members and friends. This was pointed out as a major feature of the sport. The senior orienteering athletes also regarded orienteering as a unique sport in that it promotes inter-generational social interaction. Orienteers between 4 and 90 years old are commonly competing at the same events. One woman described how sharing a mutual interest makes age less important something that you even tend to forget about in social encounters.

*“I often think when I talk to and meet [people] at orienteering competitions, and it doesn’t even need to be at orienteering events, it can be wherever, when you meet someone and then I remember after a while-oh god this is not really my friend its her parents that are my friends and this could be someone that is forty years old or something and then I realize that we had just as much fun when we stood there talking, because we have lots to talk about and then you forget about age”* (The female group)

One of the male orienteers also made a statement on the same subject, talking about how the age becomes unimportant when you can practice the same sport and compete on the same terms.

*“As an 80 year old, you feel almost the same age as a 10 year old because you can do the same things [referring to orienteering]”* (The male group)

The male athletes also discussed social interaction as a reason for staying engaged with your sport, and also brought up the importance of *being part of a community,* referring to the importance of doing things together and belonging to a club. Orienteering was further uniformly described as unique in that it is a *lifelong sport,* particularly promoting prolonged continuous engagement, as there is no age-limit to its practice.

### Follow-up question–phase 3: OR

A follow-up question addressing the participants’ objective in staying physically active in old age was developed based on the FGD findings and sent out to all participants from phase 1 of the data collection. Out of the 122 subjects, 88 completed and returned the questionnaires, resulting in a 72.1 % response rate.

The multiple choice question illustrated that the most important reason to stay physically active among senior orienteering athletes is the *improvement of physical health,* while the least important factor was found to be *perform well at competitions*. Furthermore, *improvement in mental health* was reported as being a stronger motivator to staying physically active than *being part of a social context*. It should, however, be noted that the majority of the orienteers reported all offered alternatives as equally important for their motivation to staying physical active, except for *performing well at competitions* which was substantially less frequently chosen than the other response options.

## Discussion

This mixed-method study was designed to explore and characterize active senior orienteering athletes as a potential model of healthy aging and is, to our knowledge the first of its kind.

Today, the older population is steadily growing and will, in the future, consume the majority of health care resources. It will, therefore, be important for society and health care systems to develop strategies to support older adults and increase the number of healthy years that they have. As a first step to developing new strategies for promoting health in the older population, it is essential to investigate and explore the healthy aging process. In the current paper senior orienteering athletes were thoroughly characterized, as a first step in identifying factors important for maintaining health in the later years of life.

Orienteering is an endurance-running sport involving navigation with the help of a map and a magnetic compass. The competitions are designed as a timed individual race. Obligatory control points are indicated on the map, often covering large differences in altitude and terrain conditions. The locations of the control points are unknown to the orienteers until the competition starts. The orienteer completing the correct course in the shortest amount of time is the winner. Thus, orienteering is a sport that requires both excellent physical health and considerable cognitive skills. It is these two features, in combination with the large number of aged individuals who perform the sport that make orienteering particularly interesting from a healthy aging perspective.

As outlined in the quantitative results section, we identified a significantly better health status in the senior orienteering athletes compared to the free-living older adults. Not surprisingly, the senior orienteering athletes were found to perform physical activity to a significantly greater degree when compared to the free-living older adults. Furthermore, we identified no differences in physical activity in either cohort between the summer and winter seasons as judged by the FGAS, suggesting that the activity level of older adults does not increase during the summer and spring even though the weather is better and the streets are more accessible.

Six participants of the 122 in the OR population were found to score a physical activity level of 3 on a 6-graded scale, a level corresponding to a lighter level of physical activity of 2-4 h per week. This could be interpreted as a remarkably low level of physical activity among this population. To further evaluate if the low physical activity score corresponded to an overall diminished health status, all quantitative data from these study participants were extracted and are illustrated in Additional file 1. For three of these subjects, the low level of physical activity was found to correspond to scores below the IQR 25 % percentile on the EQ-VAS and HI health measure, respectively, as seen in Additional file 1. The other three study participants reported scores within the IQR range on all questionnaires and thus, their low physical activity score did not correspond to an overall diminished health status.

It should, however, be noted that the FGAS scale provides only a rough estimate of physical activity level. Using a more fine-tuned instrument, such as the International Physical Activity Questionnaire (IPAQ) would have given us more information regarding the two populations physical activity level as well as inactivity- information that may have increased our understanding particularly with regard to the low physical activity scores reported by six of the OR athletes. A more fine-tuned instrument may have also been able to detect differences in activity level between the summer and winter seasons. However, in a pilot study, we found that the IPAQ questionnaire was not suitable for older adults as they had difficulties remembering the amount of time they spent doing different activities each day.

Our results further show that senior orienteering athletes experience a significantly greater subjective wellbeing compared to free-living older adults, as measured by the EQ-5D-5 L. One orienteering athlete was, however, found to score at a low level of 50 % on the EQ-VAS scale. In this case, the low EQ-VAS score correlated to problems with pain, dizziness, sleeping and feelings of fatigue and loneliness, as measured by the functional health measure HI, and to an elevated anxiety level, as judged by HADS. Thus, the low EQ-VAS score reported by this particular person may be attributed to these concomitant complaints, which are likely to be independent from the practice of orienteering.

Even though a significantly greater subjective wellbeing was identified among the OR population compared to free-living older adults, as judged by the EQ-5D-5 L, it is important to note that the medians of the EQ-5D index were the same for the two populations and, hence, there was no clear difference between the two groups, indicating that the majority of individuals in both populations experience a high level of subjective wellbeing. However, we did identify a higher proportion of subjects with a lower subjective wellbeing among the free-living older adults (13.5 %) compared to the senior orienteering athletes (0.82 %), as judged by EQ-VAS.

In addition to EQ-5D-5 L, several other instruments measuring subjective wellbeing do exist. The Short Form (SF)-36 is a more comprehensive instrument than the EQ-5D-5 L and includes an additional section on energy. Moreover, SF-36 has been developed based on the World Health Organization’s definition of health, which is described as physical, psychological and social wellbeing and not merely the absence of disease or infirmity. Thus, this instrument covers health from a broader perspective compared to the EQ-5D-5 L. By using a more fine-tuned instrument, we may have been able to identify more differences between the groups. However, when testing the applicability of the SF-36 among older adults we found that the questionnaire was difficult for the study participants to comprehend-particularly the section on functionality. We, therefore, decided to use an instrument less complicated but still validated and commonly used internationally to evaluate subjective wellbeing.

Furthermore, the level of depression and anxiety was found to be the same between senior orienteering athletes and free-living older adults. Among the nine orienteering athletes scoring high on HADS, two were found to score at a level indicative of anxiety or depression (≥11) [25]. One of the two was identified as the participant also scoring low on the EQ-VAS scale, as mentioned. In addition, the nine participants scored outside of the IQR range of the total OR population on several of the questionnaires, as shown in Additional file 2. The individuals reported, for example, problems with pain and elevated psychological distress on the EQ-5D index and sleep deprivation, tiredness and pain on the HI scale. This suggests that they suffered from a partly diminished health status, which did not correlate with a low level of physical activity.

Notably, we observed a significantly lower frequency of gastrointestinal problems among the senior orienteering athletes. Particularly, the fact that only 9.8 % of the senior orienteering athletes reported moderate gastrointestinal symptoms compared to 42.1 % of the free-living older adults is striking as gastrointestinal problems are a widespread phenomenon among older adults, especially among nursing home residents [26]. A connection between the gut microbiome composition and acceleration of health deterioration in aging, mediated by life-style and diet, has recently been described by Claesson et al. [27]. Thus, further emphasizing senior orienteering athletes as a highly interesting population to study when considering changes in gut microbiome composition and its relation to health and immune function in older adults. Investigating biological markers in this population will be the next step in further characterizing the healthy aging process.

Moreover, the HI questionnaire, measuring the general health status, also showed a significantly better score among the senior orienteering athletes compared to the free-living older adults, confirming our overall findings that senior orienteering athletes experience a greater physical and subjective health compared to free-living older adults.

When interpreting our results, it is important to consider that the reference values are obtained from free-living older adults, still living independently in ordinary housing, not yet in need of care at nursing homes, and therefore, representing a rather well-functioning and healthy sample of the older population. This is an essential group to include when investigating healthy aging, as these older adults will in the near future be in need of elevated health care resources. Furthermore, it is also within this group that one might want to consider introducing public health interventions, in order to help older adults maintain a high degree of health throughout life and limit their need for care in nursing homes and hospitals. It is, therefore, interesting to note that, compared to this relatively well-functioning population, we still identify a better health in the OR study population.

It should, however, be noted that our results are based on self-reported data and thus rely on the respondents’ honesty, accurate understanding and interpretation of the questions asked. Neither can we neglect the possibility that the performed study might have attracted orienteering athletes that are feeling well and experience good health. Thus, we cannot exclude the possibility that our results are skewed and represent a higher health status among the OR study population than is the case among the general population of older adults practicing orienteering in Sweden. In addition, since this study was designed to explore senior orienteering athletes as a potential model of healthy aging, it was cross-sectional in nature and, therefore, causal relationships cannot be determined. In future studies, it will be of great importance to conduct a longitudinal study to investigate the relationship between health status, behaviors and opinions over time in this particular group of older adults.

Even though questionnaire data, in general, has limitations, it is important to point out that the results from the FGDs confirm our quantitative findings that the orienteers experience a high degree of wellbeing and health. In addition the FGDs identified several factors motivating continuous engagement in physical activity.

According to the LO population, experiencing health means having the ability to perform physical exercise, being outside and enjoying nature, being able to live the life you desire, and feeling physically and mentally strong. They also stated that one can experience health when you eat tasty food, engaging in cultural pursuits (such as going to the theater or reading books) and being socially engaged. Furthermore, the orienteers suggested that one needs to be physically active, maintain, a positive attitude, have regular checkups, and avoid unhealthy environments and habits in order to maintain health. They also pointed out that gut-function has a great impact on the health status.

Moreover, the LO population consistently expressed that being a senior orienteer provides a social context and an environment that is fulfilling and supports the maintenance of physical and mental health. Previous work by our research group has identified these factors in particular as essential in order to experience wellbeing and satisfaction in old age [2]. Taken together, our results show that this aged population of orienteers embodies a preferable manner of aging, with well-maintained physical and emotional health as well as few prescribed medications. Our findings support our hypothesis and show that senior orienteering athletes may be regarded as a potential model of healthy aging.

However, it is important to point out that the qualitative data were obtained from a small sample, recruited from a small region. Furthermore, the sample of FGDs could be seen as selective, as it might have been the “healthiest” athletes who attended. Thus, we cannot exclude that this may affect the representativeness of our findings in relation to the OR cohort. Furthermore, the qualitative data presented here may not reflect the experience of senior orienteering athletes in general in Sweden.

The orienteers further emphasized that it is of important to have a balance between pleasure and physical exercise. According to the orienteers, physical exercise must be driven by passion and enjoyment, and the body and muscles must be carefully looked after. This highlights an important perspective on health in relation to physical activity and is particularly interesting, as an excess of physical exercise and pressure have been found to jeopardize both physical [28] and psychological health [29]. Previous studies have reported that highly active athletes have an increased susceptibility to upper respiratory tract infections [30, 31] in combination with lower levels of protective saliva Immunoglobulin A [30, 32]. Thus, it may not be preferable to be highly active in old age, but rather, to find a balance between enjoyment and physical activity. Perhaps the uniqueness of orienteering is that you do not have to overexert yourself and you can find a balance that fits your personality. Taken together, these findings may favor healthy aging models with cohorts that are not excessively active.

It is also important to note that the social atmosphere surrounding orienteering is considered quite unique. Orienteering is largely performed as a family sport, i.e. orienteers usually have parents and/or children practicing orienteering, and therefore the sport is strongly associated with social engagements [33]. In the present study, the LO orienteers particularly expressed the satisfaction of being able to share their sport with their family members (i.e. spouse, children and grand children) and the joy it brings them to be part of a sport in which sportsmen from such wide age ranges as 4 to 90 years old compete and socialize at the same events. They emphasize that this makes them feel young and share a connection with their co-competitors no matter their age. This differentiates orienteering from other sports, such as cycling, which is usually performed individually or with friends and is usually not shared across generations in the family.

Furthermore, the orienteers’ enjoyment in interacting with individuals across generations is of particular interest, as a few successful examples exist in Sweden in which nursing homes have been joined with daycare centers. At these combined centers, children and older adults perform physical activity together and enjoy the outdoors. According to our findings, it may be beneficial to continue this trend.

Moreover, the combination of social interaction, physical endurance and cognitive skills involved in orienteering is reflective of the three main components of Successful Aging (SA) as described by Rowe and Kahn [34, 35]: 1) avoiding disease and disability by 2) maintaining high physical and cognitive function and 3) continuously engaging in social and productive activities. Today, the majority of research on healthy aging is focused toward either biomedical or psychosocial approaches and the lay perspectives are rarely considered. These traditional approaches are becoming increasingly inadequate and we are in need of a comprehensive individual-oriented model to face the growing challenge of health-maintenance in the aging population. As outlined in the current study, senior orienteering athletes represent an interesting cohort of successful agers that may aid in this purpose. As these older sportswomen and men already master the purposed components of SA and have the prerequisites and motivation to continue with their healthy way of life, the likelihood of maintaining successful aging until a very old age is high.

The LO orienteers interviewed in this study described orienteering as a “lifelong” sport, supported by the median of 45.5 (34–55.8) years that the study participants have practiced orienteering. The lifelong engagement in orienteering further highlights the importance of physical activity throughout life as an essential factor in maintaining a healthy lifestyle in old age. The majority of the orienteers expressed in the FGDs that they grew up in an environment supporting physical activity, either by having active parents, enjoying schools sports or being influenced by friends. Thus, our results also emphasize the importance of motivating children to engage in sports from a young age, illustrating the importance of physical education in schools as an arena for children to get more acquainted with various sports and activities. In addition, the long-term engagement in orienteering further emphasizes the sound climate surrounding the sport as one can practice orienteering throughout the major part of one’s life. In support of this, orienteers have previously been found to adopt a healthier lifestyle and experiences a considerably low frequency of illness compared to the general population [33].

Even though the orienteering athletes are well aware of the long-term benefits of physical activity and their engagement in the sport was initiated in early life, it is interesting to note that they still emphasize that physical activity is driven by enjoyment and passion. They also point out that the social context is essential in order to experience health. Thus, the importance of short-term values should not be neglected as a motivator when introducing a healthy life-style in old age. Based on our findings it would be interesting in future studies to identify individual preferences (i.e. short-term values) which make an activity meaningful and rewarding, and investigate whether these can act as motivators to initiating healthy routines and making healthy choices among the general population of older adults.

## Conclusions

Our mixed-method results suggest that senior orienteering athletes may act as an ideal model of healthy aging. The orienteering sport provides its senior practitioners with a unique opportunity to practice and strengthen physical and cognitive abilities while enjoying a common interest shared with family and friends of a wide age range. In addition, the population of senior orienteering athletes was identified as a homogeneous sample (in terms of health status, engagement in physical activity, use of medication and the nonexistence of smoking habits), which further facilitates studies on healthy aging as the heterogeneity in cohorts of older adults commonly creates difficulties in performing research on persons within this age range [8]. Thus, senior orienteering athletes represent a population of healthy older adults from which we can learn how to maintain health throughout life. Furthermore, we conclude that the senior orienteering athletes’ healthy life-style is commonly founded during childhood. Thus, the importance of an early introduction of physical activity and health education cannot be neglected, in order to maintain a healthy life style throughout life. Yet, the senior orienteering athletes state that their continuous engagement in orienteering is driven by short-term values such as enjoying being outside in nature, sharing the interest with family and friends, and feeling physically and mentally strong. These findings may be of importance when considering public health interventions among the general older population.

## Additional files

Additional file 1:
**Six participants of the 122 in the OR population were found to score a physical activity level of 3 on a 6-graded scale, a level corresponding to a lighter level of physical activity of 2–4 h per week.** This could be interpreted as a remarkably low level of physical activity among this population. To further evaluate if the low physical activity score corresponded to an overall diminished health status, all quantitative data from these study participants were extracted and are illustrated in Additional file 1. For three of these subjects, the low level of physical activity was found to correspond to scores below the IQR 25 % percentile on the EQ-VAS and HI health measure, respectively, as seen in Additional file 1. Additional file 2:
**Among the nine orienteering athletes scoring high on HADS, two were found to score at a level indicative of anxiety or depression (**≥**11) [** 25 **].** One of the two was identified as the participant also scoring low on the EQ-VAS scale. In addition, the nine participants scored outside of the IQR range of the total OR population on several of the questionnaires, as shown in Additional file 2. The individuals reported, for example, problems with pain and elevated psychological distress on the EQ-5D index and sleep deprivation, tiredness and pain on the HI scale. This suggests that they suffered from a partly diminished health status, which did not correlate with a low level of physical activity.

## References

[1] Cohen JE (2003). Human population: the next half century. Science.

[2] Algilani S, Ostlund-Lagerström L, Kihlgren A, Blomberg K, Brummer RJ, Schoultz I (2014). Exploring the concept of optimal functionality in old age. J Multidiscip Healthc.

[3] Rattan SIS (2004). Aging, anti-aging, and hormesis. Mech Aging Dev.

[4] Grad FP (2002). The preamble of the constitution of the world health organization. Bull World Health Organ.

[5] Adler RH (2009). Engel’s biopsychosocial model is still relevant today. J Psychosom Res.

[6] Engel GL (1977). The need for a new medical model: a challenge for biomedicine. Science.

[7] Engel GL (2012). The need for a new medical model: a challenge for biomedicine. Psychodyn Psychiatry.

[8] Cevenini E, Invidia L, Lescai F, Salvioli S, Tieri P, Castellani G, et al. Human models of aging and longevity. Expert Opin Biol Ther. 2008;8:1393–405. 10.1517/14712598.8.9.1393 18694357

[9] Franceschi C, Motta L, Valensin S, Rapisarda R, Franzone A, Berardelli M, et al. Do men and women follow different trajectories to reach extreme longevity? Italian Multicenter Study on Centenarians (IMUSCE). Aging (Milano). 2000;12:77–84. 10.1007/BF03339894 10902049

[10] James I, Blomberg K, Kihlgren A (2014). A meaningful daily life in nursing homes - a place of shelter and a space of freedom: a participatory appreciative action reflection study. BMC Nurs.

[11] Sandelowski M (2000). Combining qualitative and quantitative sampling, data collection, and analysis techniques in mixed-method studies. Res Nurs Health.

[12] Polit DF (2004). Nursing research: principles and methods.

[13] Krippendorff K (2013). Content analysis: an introduction to its methodology. 3^rd^ ed.

[14] Elo S, Kyngäs H (2008). The qualitative content analysis process. J Adv Nurs.

[15] Creswell J, Plano Clark V (2011). Designing and conducting mixed methods research.

[16] Frändin K, Grimby G (1994). Assessment of physical activity, fitness and performance in 76-year-olds. Scand J Med Sci Sports.

[17] Brauer CA, Rosen AB, Greenberg D, Neumann PJ (2006). Trends in the measurement of health utilities in published cost-utility analyses. Value Health.

[18] Rabin R, de Charro F (2001). EQ-5D: a measure of health status from the EuroQol Group. Ann Med.

[19] Forsberg C, Björvell H (1993). Swedish population norms for the GHRI, HI and STAI-state. Qual Life Res.

[20] Svedlund J, Sjodin I, Dotevall G (1988). GSRS–a clinical rating scale for gastrointestinal symptoms in patients with irritable bowel syndrome and peptic ulcer disease. DigDisSci.

[21] Snaith RP, Zigmond AS (1986). The hospital anxiety and depression scale. BrMedJ(ClinResEd).

[22] Bjelland I, Dahl AA, Haug TT, Neckelmann D (2002). The validity of the Hospital anxiety and depression scale. An updated literature review. J Psychosom Res.

[23] Kitzinger J, Barbour RS (1999). Developing focus group research: politics, theory, and practice.

[24] Sørensen J, Davidsen M, Gudex C, Pedersen KM, Brønnum-Hansen H (2009). Danish EQ-5D population norms. Scand J Public Health.

[25] Snaith RP (2003). The hospital anxiety and depression scale. Health Qual Life Outcomes.

[26] Bosshard W, Dreher R, Schnegg JF, Bula CJ (2004). The treatment of chronic constipation in elderly people: an update. Drugs Aging.

[27] Claesson MJ, Jeffery IB, Conde S, Power SE, O’Connor EM, Cusack S, et al. Gut microbiota composition correlates with diet and health in the elderly. Nature. 2012;488:178–84. 10.1038/nature11319 22797518

[28] Lehmann MJ, Lormes W, Opitz-Gress A, Steinacker JM, Netzer N, Foster C, et al. Training and overtraining: an overview and experimental results in endurance sports. J Sports Med Phys Fitness. 1997;37:7–17. 9190120

[29] Smith RE (1984). The dynamics and prevention of stress-induced burnout in athletics. Prim Care.

[30] Gleeson M, McDonald WA, Cripps AW, Pyne DB, Clancy RL, Fricker PA (1995). The effect on immunity of long-term intensive training in elite swimmers. Clin Exp Immunol.

[31] Gleeson M, Bishop N, Oliveira M, McCauley T, Tauler P, Muhamad AS (2012). Respiratory infection risk in athletes: association with antigen-stimulated IL-10 production and salivary IgA secretion. Scand J Med Sci Sports.

[32] Mackinnon LT, Hooper S (1994). Mucosal (secretory) immune system responses to exercise of varying intensity and during overtraining. Int J Sports Med.

[33] Ottosson T (1996). Swedish orienteers: a survey study. Sci J Orienteering.

[34] Rowe JW, Kahn RL (1997). Successful aging. Gerontologist.

[35] Rowe JW, Kahn RL (1987). Human aging: usual and successful. Science.

