# Supplementary material for: Senior orienteering athletes as a model of healthy aging: a mixed-method approach
Source: BMC Geriatr. 2015 Jul 8;15:76. doi: 10.1186/s12877-015-0072-6 (PMC4495641; doi:10.1186/s12877-015-0072-6)
Supplement: Additional file 1: — Six participants of the 122 in the OR population were found to score a physical activity level of 3 on a 6-graded scale, a level corresponding to a lighter level of physical activity of 2–4 h per week. This could be interpreted as a remarkably low level of physical activity among this population. To further evaluate if the low physical activity score corresponded to an overall diminished health status, all quantitative data from these study participants were extracted and are illustrated in Additional file 1. For three of these subjects, the low level of physical activity was found to correspond to scores below the IQR 25 % percentile on the EQ-VAS and HI health measure, respectively, as seen in Additional file 1. [file 12877_2015_72_MOESM1_ESM.pdf]

Additional file 1 Questionnaire median scores OR subjects scoring low on FGAS

Subjects	FGAS	FGAS *Summer*	FGAS *Winter*	EQ-VAS	EQ-Index	HI	GSRS	HADS
Score range:	*1-6*	*1-6*	*1-6*	*0-100*	*0-1*	*9-36*	*1-7*	*0-42*
**subject 1**	3	3	3	98[a]	1.0	28[b]	1.1	2.0
**subject 2**	3	3	3	90	1.0	35[a]	1.1	1.0[b]
**subject 3**	3	3	3	75[b]	0.8	29	1.1	2.0
**subject 4**	3	3	3	80[b]	0.9	32	1.4	5.0
**subject 5**	3	3	3	85	1.0	33	1.3	1.0[b]
**subject 6**	3	3	3	90	0.8	31	1.1	4.0

a: Values scored above the 75% percentile, b: Values scored below the 25% percentile
