# Supplementary material for: Senior orienteering athletes as a model of healthy aging: a mixed-method approach
Source: BMC Geriatr. 2015 Jul 8;15:76. doi: 10.1186/s12877-015-0072-6 (PMC4495641; doi:10.1186/s12877-015-0072-6)
Supplement: Additional file 2: — Among the nine orienteering athletes scoring high on HADS, two were found to score at a level indicative of anxiety or depression (≥11) [ 25 ]. One of the two was identified as the participant also scoring low on the EQ-VAS scale. In addition, the nine participants scored outside of the IQR range of the total OR population on several of the questionnaires, as shown in Additional file 2. The individuals reported, for example, problems with pain and elevated psychological distress on the EQ-5D index and sleep deprivation, tiredness and pain on the HI scale. This suggests that they suffered from a partly diminished health status, which did not correlate with a low level of physical activity. [file 12877_2015_72_MOESM2_ESM.pdf]

Additional file 2 Questionnaire median scores for OR subjects scoring high on HADS

Subjects	HADS	HADS *Depression*	HADS *Anxiety*	EQ-VAS	EQ-Index	HI	GSRS	FGAS
Score range:	*0-42*	*0-21*	*0-21*	*0-100*	*0-1*	*9-36*	*1-7*	*1-6*
**subject 7**	11	3	8[a]	70[b]	0.79[b]	33	1.6	6
**subject 8**	16	6[a]	10[a]	90	0.73[b]	-	2.1[a]	5.5
**subject 9**	12	2	10[a]	50[b]	0.69[b]	22[b]	1.8[a]	4
**subject 10**	19	12[a]	7[a]	80[b]	0.86	27[b]	1.4	4
**subject 11**	13	4[a]	9[a]	90	0.73[b]	26[b]	1.8[a]	4.5
**subject 12**	14	6[a]	8[a]	90	0.79[b]	27[b]	1.5	5
**subject 13**	11	2	9[a]	75[b]	0.77[b]	25[b]	2.7[a]	5
**subject 14**	13	5[a]	8[a]	80[b]	0.80	27[b]	1.8[a]	4
**subject 15**	14	3	11[a]	75[b]	0.77[b]	28[b]	1.8[a]	4

a: Values scored above the 75% percentile, b: Values scored below the 25% percentile
